# Breaking bad news to antenatal patients with strategies to lessen the pain: a qualitative study

**DOI:** 10.1186/s12978-018-0454-2

**Published:** 2018-01-23

**Authors:** José Atienza-Carrasco, Manuel Linares-Abad, María Padilla-Ruiz, Isabel María Morales-Gil

**Affiliations:** 1 Nursing Surgery Service, Hospital Costa del Sol, Málaga, Spain; 2 School of Health Sciences, Universidad de Jaén, Jaén, Spain; 3 Research Unit. Agencia Sanitaria Costa del Sol, Marbella, Spain; 4 Health Services Research on Chronic Patients Network. REDISSEC, Madrid, Spain; 5 School of Health Sciences, Universidad de Málaga, Málaga, Spain

**Keywords:** Communicative skills, Prenatal diagnosis, Congenital anomalies, Pregnancy, Interpersonal relations, Qualitative research

## Abstract

**Background:**

To consider the thoughts and actions of healthcare personnel in situations when an adverse prenatal diagnosis must be communicated, including appropriate strategies and skills to respond to information needs and to manage the emotional responses of patients.

**Methods:**

Descriptive qualitative study using non-participant observation and semi-structured interviews to analyse the discourses of physicians, midwives, nurses and nursing assistants who provide healthcare to obstetric patients.

**Results:**

There may be barriers to effective communication between healthcare personnel and patients, depending on the characteristics of the persons involved, the organisation of healthcare, biotechnological progress and cultural factors.

**Conclusions:**

The human quality of healthcare has deteriorated due to excessive workloads and to the growing role played by technology. In order to improve communication, more attention should be paid to human and spiritual dimensions, prioritising empathy, authenticity and non-judgmental listening. An appropriate model of clinical relationship should be based on shared decision making, clarifying the functions of the multidisciplinary team to alleviate a mother’s suffering when a pregnancy is interrupted. To do so, protocols should be implemented to ensure the provision of comprehensive care, not only addressing biological issues but also providing psychosocial attention. Finally, training should be provided to healthcare staff to enhance their social skills and cultural competence.

This study identifies potential improvements in the interventions made by healthcare personnel and in the organisation of the institution, concerning the attention provided to pregnant women when an adverse prenatal diagnosis must be communicated.

## Plain English summary

Although scientific and technological progress has provided major health benefits, it can also hamper communication between healthcare personnel and patients. In modern medicine, the technical aspects of healthcare tend to prevail, in which the main focus is placed on treating the disease and less attention is paid to other aspects that are also important to the patient, such as feelings and emotions. Knowing how to communicate is an ethical and legal imperative and therefore healthcare professionals must ensure that patients are aware of everything related to their condition, to facilitate their autonomy in decision-making. If knowing how to communicate is always important, it is even more so when the content of the message is unfavourable. For example, bad news about the advance of a pregnancy can influence the mother’s decision on whether to continue or to interrupt it. Professional interventions in such cases are crucial, because the psychological consequences of the situation depend on the care and support provided. Deficiencies in the communication process can generate conflicts and dissatisfaction in the professional-patient-family relationship.

## Background

Technological progress and advances in the field of molecular genetics have contributed decisively to the development of prenatal diagnostics. Biotechnology offers novel, highly reliable instruments and techniques for identifying maternal risk factors, enabling the early detection of congenital malformations or defects of diverse types related to foetal formation and development [1]. However, despite this considerable progress, there is often a profound lack of interest in interpersonal communication. The ability to communicate with patients has been erroneously viewed as a lesser skill, compared to technical aspects of healthcare [2, 3], when in fact it is an essential element in the relationship between healthcare personnel and their patients, based on mutual recognition and shared decision making [4–6].

When a chromosome defect or severe foetal malformation is confirmed, the health team is obliged to inform the mother about the advisability of continuing the pregnancy, and of the prognosis and possible postpartum outcomes [7–10] respecting the patient’s right to choose [4]. In many cases, healthcare personnel develop their own strategies, without taking into account the patient’s holistic nature and without forming a comprehensive outlook on the health-disease binomial, capable of transforming information into a therapeutic tool [11]. Carers tend not to listen, thereby failing to obtain feedback to clarify exactly what the patient requires, and becoming emotionally distant. This inability to adapt the communication of diagnostic information to the patient’s own values and preferences can generate conflicts and dissatisfaction [11–14]. Attitudes and communicative skills play a fundamental and decisive role in addressing problems, helping patients overcome psychological distress and conveying realistic expectations [15]. In prenatal preventive care programmes, the evaluation of obstetric risk, as well as ruling out biologically-based problems and identifying women at high risk of maternal and perinatal complications, should also address the patient from an inclusive perspective, because (among other reasons) the health-disease process is a multidimensional one in which biological, psychological and social factors continuously interact, in a positive or a negative sense [16].

The aim of this study is to examine the healthcare provided to pregnant women whose foetuses present congenital defects and to facilitate the design of a more personalised health model, one that responds to all their needs, rather than a limited selection. Accordingly, we have analysed the views expressed by healthcare personnel and the interventions they make when an adverse prenatal diagnosis must be communicated, including the strategies and skills employed to meet patients’ information needs and to respond to their emotional responses.

## Methods

This qualitative study was undertaken from a phenomenological standpoint, which enabled us to analyse specific experiences [17]. Information was collected by means of non-participant observation and semi-structured interviews, obtaining opinions from physicians, midwives, nurses and nursing assistants involved in real-life care processes relevant to the object of our study. Our analysis is based on descriptive and interpretative paradigms, which were applied to interpret the data obtained in the context of existing knowledge about the study area and in that of the participants’ experiences in this respect. To maximise the scientific and methodological rigour of the study, criteria of credibility, transferability, dependence (or consistency) and confirmability were applied [17, 18].

The study was carried out at the Costa del Sol Health Agency (Marbella, Spain) from June to September 2015. During the information-compiling phase, 37 interviews were conducted, with 22 obstetricians, four midwives, three nurses and eight nursing assistants (Table 1). These healthcare personnel all worked in units within the Obstetrics service (prenatal diagnosis, perinatal medicine, hospitalisation, first, second and third-trimester pregnancy consultation and/or obstetric ambulatory care). The selection of the participants was intentional and not random, taking into account the criterion of segmentation, depending on the occupational category, hospital unit, position occupied and duration of employment at the hospital. The inclusion criteria were that participants should be physicians, midwives, nurses or nursing assistants at the hospital, have at least 1 year’s experience (to acquire sufficient knowledge of the unit) and give permission for audio recording of the interview. All potential participants were sent letters indicating the purpose of the study and inviting them to take part. Participation was later confirmed by telephone.

**Table 1:** Sociodemographic characteristics of healthcare professionals

Professional category	Obstetrician	Midwife	Nurse	Nursing assistant
Sex				
Male				
Age				
<40	2	1		
41–45				
46–50				
>50	2			
Female				
Age				
<40	11	2	2	3
41–45	5	1		2
46–50	2		1	1
>50				2
Total	22	4	3	8
Mean age (Years)	39	38	40	44
Mean Experience (Years)	11	10	16	24

The principal investigator^1^ directly observed the interactions between patients and healthcare personnel, paying attention both to their words and to non-verbal aspects. A field diary was used to make a detailed record of all observations, after patients were informed and gave their written consent to the procedure. To avoid subjectivity, a feedback process was also applied, in which the notes made and impressions received were later shared with the same participants during the interviews.

The script for the interviews was elaborated ad hoc, taking into account our previous review of the literature and the dimensions of the study, and was examined and agreed upon by all members of the research team. The recordings were transcribed and NVivo 11 qualitative software was used to encode the information and to perform the content analysis. The qualitative data analysis was carried out using the Taylor-Bogdan system, based on data preparation, identification of emerging issues, coding, interpretation, relativisation and determination of methodological rigour [19, 20].

Both emerging issues (those arising during the interview) and predefined ones (discussion topics included in the interview design) were identified. To ensure optimum data quality, triangulation was applied, regarding both the data (using data compilation instruments such as interviews and non-participant observation) and the researchers (the analysis was performed by two researchers^1,3^, first working independently and then reaching a consensus view) [21].

Data saturation was determined after analysing the number of references encoded and dimensions identified, as the point at which the reading and coding process failed to generate additional information that would require further codes or categories.

## Results

Data analysis revealed the existence of three related categories: how to give bad news, communication skills in general and interactions between healthcare professionals and patients.

The following results are presented in terms of the corresponding information category, highlighting the main findings obtained for each subcategory. These findings are also shown in the tables, together with verbatim transcripts from the interviews. The occupation of the healthcare professional is identified by a code assigned to the interview, thus ensuring anonymity and confidentiality. Finally, the data obtained are considered in the light of previous research reports in this regard.

### How to give bad news

#### Issuers and receivers of bad news

Healthcare workers emphasise the unexpected nature of bad news and the unwanted changes they provoke in the lives of those who receive it.

Regarding who should perform this communication, there is general agreement that this should be the obstetrician, without excluding the support that other team members may provide, as long as they are well informed about the case. All accept that the pregnant woman should be the first to receive the message, that her autonomy as a human being should be recognised and that she has the right to be informed about everything related to her own health and to that of the foetus. Obstetricians stress how important it is to inform the woman’s partner, too, from the outset or, if this is impossible, a person of trust who can provide support and liaison between the parties.

With respect to the medical consultation, the general opinion of the participants is that the person accompanying the mother (usually, the partner or a close relative) should have the patient’s consent to hear the details about the evolution of pregnancy, as part of the clinical relationship. None of the healthcare workers believe that information should be concealed or that any pact of silence should be made with the family.

#### What bad news is communicated and how

Among the most frequent areas of bad news mentioned are diagnoses of miscarriage, chromosomal alteration and intrauterine foetal malformation or death. According to the participants, the early detection of congenital anomalies, in prenatal diagnosis, usually means that a decision must be taken on whether or not to interrupt the pregnancy.

Regarding how it is communicated, from the standpoint of the healthcare professionals, it is advisable to give immediate notice of the discovery of bad news and to inform the patient of the alternatives available. Whatever the prognosis, the availability of equipment to control the symptoms and to ensure the best possible quality of life and comfort should be stressed.

Most of the participants agreed that information should be transmitted gradually, and if possible in line with the patient’s wish to receive it. According to healthcare workers, the patient’s first reaction is often to suffer a state of shock, in which she is unable to process the information provided. A nursing assistant who works in pregnancy monitoring consultations and who, moreover, has a degree in psychology, illustrates this very well with the following comment:

#### AUXE01

*“Gradually. I think that we should tell them everything, that the information must be complete, and not concealed or ignored, no matter how painful it may be. On the other hand, it mustn’t be blurted out too abruptly. All extremes are bad; being told little by little might be agonising, but all at once can be devastating. It’s important to enable feedback; you tell them the situation and, depending on the reactions you get, you know where to place more emphasis and where you need to tread lightly.”*

Regarding how the information should be provided, some professionals choose to prepare the ground before entering fully into the question, others prefer to convey optimism and to leave a door open to hope during the first contact with the patient. In any case, for those interviewed the communication of bad news is not a “single or isolated act” that ends here and now, but must be viewed as a process that requires time and effort. The healthcare team believes it important to inform the person affected that there will be more meetings and opportunities for her to express doubts that may arise, or simply her pain and anger.

The healthcare professionals consulted in our study consider it important to avoid the excessive use of technical terms and to adapt their verbal language to the cultural level of the patient, as detected at the time of the interview. Among the notes made in field diaries during the observation, it is significant that negative reactions were aroused among patients by certain expressions commonly used in obstetrics terminology, such as miscarriage, malformation or foetal death. In this respect, opinions among the professionals are divided: the majority consider that such terms must be used, in order to avoid ambiguity, but others prefer to use alternative expressions, in order to soften the emotional impact produced.

Within this subcategory, an aspect of some importance is that of non-verbal language in interactions with the patient. The researchers noted a reaction that was also observed by many of the obstetricians consulted, namely that the non-verbal language used by healthcare professionals during the ultrasound exploration produces expectation and concern in the women being examined.

#### Optimum environmental conditions when bad news must be given

Noise and interruptions are the main barriers to establishing a climate of trust during meetings with patients. The healthcare workers commented that they often have to improvise a space in which to hold these meetings, since no purpose-designed physical location is reserved for this type of communication.

These professionals agree that the ideal place for the communication of bad news should provide privacy and tranquillity, be separated from the maternity area, be comfortable and have enough natural light.

#### The evolution of carer-patient communication

According to the professionals interviewed, scientific-technological advances, the increasing presence of women in obstetrics and gynaecology teams and the practice of defensive medicine are the main factors underlying the evolution of communication and clinical relationships with patients.

#### The role of nursing staff

The obstetricians consulted acknowledge the commendable work done by nurses and auxiliaries in prenatal consultations, in perinatal medicine and during the hospital stay of women who decide to terminate their pregnancy. The support offered by these hospital workers is viewed as an essential element in achieving patients’ satisfaction and collaboration.

#### Strategy and summary

Many healthcare workers professionals commented that when significant information must be transmitted patients should be given sufficient time to relax and to consider the situation and any doubts they may have. Accordingly, they are asked to leave the consultation during this time, to allow other patients to be attended. If this period of reflection is considerable, the patient may be offered an appointment for another day, especially if crucial decisions must be taken.

According to healthcare staff, lack of time (often caused by an overload of responsibilities) is responsible for the absence of feedback on the quality of the attention provided.

#### Setting of the intervention

Sometimes the suspicion or the discovery of foetal malformation arises during a routine ultrasound exploration, or the patient may be asked to attend a consultation to be given the confirmation of a previous finding. For obstetricians, the presence or absence of prior knowledge of the diagnosis and the time available are the factors that will determine the preparation or improvisation of their discourse. In this respect, healthcare workers also recognise that taking control of the situation is more complicated and that anxiety and unrest may be provoked when the diagnosis arises unexpectedly (Table 2).

**Table 2:** Category.- How to give bad news

Subcategory: issuers and receivers of bad news Verbatim
***OBST S4*** *: “A woman who is pregnant always expects everything to go well, that the delivery will go well and that a healthy baby will be born; anything that goes wrong is bad news”.*
***MID S2*** *: “Unexpected information that causes sadness and pain and provokes a change in your life”.*
***MID S1*** *: “Something that doesn’t fit what you had expected, that spoils plans and means you have to adapt to a new situation”.*
***OBST S17*** *: “The doctor is the person who can and should communicate bad news”.*
***NASST S8*** *: “The news has to be given by the doctor and we are there to provide support”.*
***MID S4*** *: “Ideally, there should be a multidisciplinary team, including nurses and, if possible, a psychotherapist”.*
***NUR S3*** *: “Although the doctor gives the news, nurses are also involved, because the patients ask us to clarify what they don’t understand”.*
***OBST S9*** *: “Obviously, you have to tell the patient and her partner, if there is one, and then assess the advisability of informing the family”.*
***OBST S2*** *: “According to the rules on patient autonomy, the patient must always be told, and we assume that whoever is with her can also receive the news”.*
***NUR S2*** *: “The patient must be informed because she is the one who is pregnant”.*
***MID S2*** *: “It is very important that the information should also be received by someone the patient trusts (…)”*
Subcategory: what bad news is communicated and how Verbatim
***NASST S6*** *: “(...) miscarriages, foetal malformations, syndromes, foetal heart disease, proposal to interrupt the pregnancy”.*
***NUR S3*** *: “In the ward, we have patients who decide to abort because of a diagnosis of severe malformation or chromosomal alteration”.*
***MID S12*** *: “Very often, non-viable pregnancies, anomalies detected by ultrasound scan, antepartum deaths, intrauterine growth problems”.*
***OBST S8*** *: “There is no magic formula. Usually the news is given little by little so that the information can be assimilated, but this does not always work, and for some patients it is very painful. Although for others, telling it all at once can be devastating (...)”*
***OBST S13*** *: “(...) using the same means seen to be successful when done by more experienced colleagues”.*
***MID S3*** *: “You create your own style, by doing it over and over again”.*
***OBST S13*** *: “(...) I prefer to be totally frank. Sometimes, only when you say that the foetus is dead or that its situation is incompatible with life does the patient realise the gravity of the news”.*
***NUR S3*** *: “We try to take into account the patient’s socio-cultural level, but we often forget and use too much medical jargon”.*
***NASST S4*** *: “(...) we aren’t very close to the patients, we don’t take their hands, we don’t give them a hug, we avoid looking directly into their eyes, we focus on filling in the report, on the computer and on the ultrasound scan”.*
Subcategory: optimum environmental conditions when bad news must begiven Verbatim
***NUR S1*** *: “Somewhere private, without interruptions, separated from the maternity area, comfortable and with sufficient natural light”.*
***OBST S13*** *: “(...) knocking at the door, people coming in and out, telephones ringing continually”.*
***MID S2*** *: “The intentions are good, but there is no area specially equipped for this purpose. We try to assign a single room for a pregnancy termination, but it is not always possible”.*
Subcategory: the evolution of carer-patient communication Verbatim
***OBST S8*** *: “We always have to bear in mind the question of the medical professional’s legal defence. The social situation makes this inevitable, but it makes it very difficult to provide personalised, direct treatment”.*
***NUR S2*** *: “Consent forms, signatures in duplicate, the next appointment with another healthcare professional ... all of this greatly interferes with the doctor-patient relationship”.*
***OBST S6*** *: “We pay more attention to the diagnostics, we’ve gained in technological capabilities and lost in human quality”.*
Subcategory: the role of nursing staff, according to the physician Verbatim
***OBST S11*** *: “The nurses provide very important support; they help us convey the message we want the patient to receive”.*
***OBST S1*** *: “They can provide support, but diagnosis is the doctor’s job and we have to communicate the message, even if we don’t like it”.*
***OBST S12*** *: “(...) nurses spend many hours at the patient’s bedside, so they are well aware of the patient’s fears and expectations”.*
Subcategory: strategy and summary Verbatim
***OBST S16*** *: “The extra time you give to one patient is time you’re taking away from another. Ideally, an appointment should be made for another day”.*
***NASST S7*** *: “Patients are seen again at the end of the consultation, to answer their questions”.*
***OBST S14*** *: “At the end of the interview I do not have enough time for the patient to repeat everything I have explained to her and check if she has understood me”.*
Subcategory: setting of the intervention Verbatim
***OBST S10*** *: “With experience, you usually know what to do, but patients can ask unpredictable questions that you have to address on the spot”.*
***OBST 16*** *: “Prior awareness or otherwise of the diagnosis and of the time available determines whether the talk to the patient can be prepared or must be improvised”.*
***OBST S13*** *: “Inadequate training in communication is a handicap, making it hard to adapt the discourse to meet all the patient’s needs”.*

*OBST* Obstetrician, *MID* Midwife, *NUR* Nurse, *NASST* Nursing assistant

### Communication skills

#### Training in the communication of bad news

The healthcare personnel who took part in this study, especially the obstetricians, commented that their academic training in communication and counselling was practically non-existent. They also observed that their acquisition of this type of knowledge was based solely on participation in a short course during initial training. Neither is specific postgraduate training provided; thus, most professionals define themselves as self-taught, having observed and learned from actions seen to be useful for other colleagues. However, these workers consider it extremely important to learn strategies that foster the creation of a solid and, above all, therapeutic clinical relationship.

Most of these professionals admit that a lack of communication techniques and social skills sometimes creates a barrier that can impede the development of a good clinical relationship. The kind of training that they consider appropriate to alleviate this problem, helping them to establish effective communication with patients and family members, would be based on periodic role-playing workshops, debriefing sessions and counselling by a psychologist.

The characteristics or skills that professionals believe should be possessed by those responsible for communicating bad news are proximity, empathy and assuredness.

#### The ability to explore psycho-social issues

Concerning the psychological aspect, we examined the extent to which healthcare professionals explore the patient’s state of mind before communicating bad news. The hypothetical case was raised that the patient was experiencing considerable stress (the death of a loved one, or a recent serious diagnosis, either personal or affecting a close family member).

Within the social sphere, we considered how professionals explore the impact of bad news on patients’ daily lives and on their families, social circles and work environments.

In response, the healthcare workers commented that no formal exploration is usually made of psychological questions or of the patient’s social sphere, in terms of a systematic examination, and emphasised that this type of inquiry does not influence the communication of bad news. Although such an investigation might be undertaken in the private sphere, these professionals consider it very difficult to do so in the public domain since the way in which services are structured and consultations planned means that there is insufficient time to conduct a formal, regulated study.

The healthcare professionals also stated that patients’ psychological and social problems should be treated in primary care and then, if appropriate, referred to a mental health clinic or to social workers.

#### Responding to the patient’s emotions

We also inquired how healthcare professionals address the emotional responses of patients who are given bad news. In general, these workers observed that providing resources to help patients adapt to the new situation, alleviate their mental pain and reduce stress, depression or anxiety is outside their field of competence as gynaecologists and obstetricians, an area in which they lack training, and therefore that this task would be undertaken more appropriately by a psychotherapist.

The professionals who took part in our study did not consider themselves well acquainted with counselling strategies or resilience models for the effective management of patients’ emotions.

Finally, the possible existence of language barriers was attributed to the cultural diversity that characterises the population of pregnant women attended at the hospital where this study was performed (Table 3).

**Table 3:** Category.- Communication skills

Subcategory: training in the communication of bad news Verbatim
***OBST S14*** *: “In the 2nd year at university, we did workshops on doctor-patient communication, but little else in the 6 years spent in the faculty.”*
***OBST S12*** *: “During the first year of residency, we had a course on the doctor-patient relationship, in which we discussed the quality of care and the communication of bad news. But nothing since then.”*
***NUR S3*** *: “(...) I’ve had training in helping and in the humanisation of care, but that was a few years ago.”*
***MID S3*** *: “Every year we offer a course on how to respond to perinatal grieving, but hardly any of the medical staff take it.”*
***OBST S13*** *: “We would need to learn communicative techniques and skills through role playing and recordings of our own interventions, and then analyse them.”*
***MOBST S1*** *: “(...) we need advice from a psychotherapist, and medical team sessions to make our criteria consistent.”*
➣Desirable qualities in the person who must transmit bad news:
***OBST S11*** *: “Sensitivity and humanity, I think.”*
***OBST S12*** *: “Empathy with the patient and showing self-assuredness in what you have to convey.”*
***OBST S6*** *: “Having sufficient knowledge of pathology, of what can and can’t be done, and time in which to carry out possible solutions.”*
***NASST S1*** *: “Closeness, putting yourself in the patient’s place and speaking in terms that she can understand.”*
Subcategory: the ability to explore psycho-social issues Verbatim
***ASST S7*** *: “This isn’t examined. Some patients will say they’ve had a stressful experience, but the doctor doesn’t go into this question, there isn’t time.”*
***OBST S2*** *: “(...) that isn’t examined. I honestly don’t know what kind of inquiry might be made. If the patient has problems of this type, she usually tells you herself.”*
***MID S2*** *: “The psychological and social aspects aren’t considered due to our feelings of insecurity. We make the excuse that we don’t have time, but it depends to a great extent on each individual’s attitude and personal interest in the matter.”*
***MID S4*** *: “Unless the patient tells you spontaneously (although you might intuitively sense it), you don’t usually go into these areas, you only address the physical side.”*
Subcategory: responding to the patient’s emotions Verbatim
***NASST S1*** *: “You don’t have the knowledge or skills to deal with certain problems and the easiest thing to do is to avoid them. Without specific and continuous training in the necessary areas, we can’t offer patients comprehensive quality care.”*
***OBST S3*** *: “We don’t have time. To respond properly we’d need a specialised consultation, with the presence of a psychologist.”*
➣Counselling strategies and Models of resilience:
***OBST S4*** *: “... yes, I’d recommend it, but to help in all these areas, right now I don’t have the tools, nor do we schedule appointments to assess the patient’s evolution, how she’s coping with the bad news or accepting it.”*
***OBST S1*** *: “I don’t know what these strategies consist of.”*
***MAT S4*** *: “What we do is listening, and little else. The patients go home, basically, with nothing.”*
➣*Language barriers:*
***OBST S2*** *: “There are language barriers, especially with the Chinese and Arab populations, and this makes you anxious.”*

*OBST* Obstetrician, *MID* Midwife, *NUR* Nurse, *NASST* Nursing assistant

### Professional-patient interaction

#### Profile of the patients

The heterogeneity of the patient population at our hospital, in terms of sociocultural status and nationality, requires healthcare professionals to be especially sensitive to the need to provide culturally appropriate care. From the statements made by the participants and from the observations made by the research team, we conclude that the sociocultural characteristics of the patients are very relevant to the communication of bad news, both in how it is received (non-verbal behaviour) and in the coping strategies then adopted.

#### Reactions to bad news

The healthcare professionals in our study population do not find it difficult to identify the emotions aroused in patients on receiving bad news, because certain patterns tend to appear repeatedly. Moreover, the professionals consider it positive to encourage the expression of these emotions.

#### Demand for information

As with emotional responses, the first questions asked by women in this situation are very familiar to healthcare workers (*“Why? What did I do wrong? Now what? Does this happen often?”).* When an adverse prenatal diagnosis is made, patients are advised to request a second medical opinion before making a final decision regarding their pregnancy, and are discouraged from seeking information on the internet, because of its unreliability. Patients are warned that the only dependable information is that provided by the health team.

#### Influence of professionals on decision-making

The professionals realise that their opinions could affect patients’ decisions about their pregnancies, depending on the communicative style employed. The way in which questions such as the prognosis and possible alternatives are addressed could facilitate or hinder a satisfactory resolution of the situation. Variability among the professionals involved in this healthcare can also make communication difficult.

#### Psychosocial support

Healthcare professionals are well disposed to accompany these patients and offer them support, but believe they lack training to respond adequately to the patients’ grieving. The general opinion is that handling difficult situations requires the intervention and support of a psychologist specialised in providing this sort of assistance.

#### Influence of technology

The professionals also referred to being stressed by the bureaucratic and treatment overload they are obliged to accept in modern treatment contexts, together with the ever-greater dependence on technology. According to the professionals, these factors prevent them from dedicating sufficient time to address human concerns and to prevent the medical act from becoming increasingly impersonal (Table 4).

**Table 4:** Category.- Patient - Healthcare professional interaction

Subcategory: profile of the patients Verbatim
***OBST S13*** *: “Young women, between 16 and 44 years old, generally healthy for pregnancy, childbirth and postnatal care. Regarding socio-cultural level, there are all types, from low socio-cultural level to middle and high levels, immigrants, Spanish natives, Asian, European, African ... a multicultural population.”*
***OBST S8*** *: “Very heterogeneous due to the variety of races.”*
***OBST S7*** *: “Because what I say may lead to the pregnancy being interrupted, it’s necessary to know that not all cultures conceive or face this prospect in the same way.”*
Subcategory: reactions to bad news Verbatim
***MID S3*** *: “At first there is a state of shock, a sense of unreality.”*
***OBST S3*** *: “They respond with pain, crying, anguish, suffering, and the feeling of enormous disappointment.”*
***OBST S8*** *: “Although the foetus referred to in the bad news is the fruit of two people, the father and the mother, the mother’s response is usually much more emotional, and the role of the father automatically becomes that of consoling the mother.”*
Subcategory: demand for information Verbatim
***NASST S6****: “Why*? *What have I done? Is it common? Finding out the cause and trying to determine if they are responsible. Is it something I took? I made an effort (...)”*
***NASST S5****: “(...) Now what*? *What can be done? What do you suggest? What would you do if it happened to you?”*
***NUR S1*** *: “I think a second opinion would be a good idea, but it is very important to know who to ask.”*
***OBST S4*** *: “They resort to the internet, but they don’t know how to filter the information and what information has been scientifically proven.”*
***MID S3*** *: “In the private sector, they think they are better looked after because they are given more time and attention. Perhaps we should improve things in this area.”*
Subcategory: influence of professionals on decision-making Verbatim
***OBST S14*** *: “(...) you are often recommend what they should do. So, the way we give the news can make their decision go one way or the other.”*
***NUR S2*** *: “The lack of social skills hinders the active participation of these patients in decision making.”*
***OBST S13*** *: “I try to be as aseptic as possible, to respect their autonomy, giving them the consent form to sign …”*
Subcategory: psychosocial support Verbatim
***OBST S10*** *: “(...) some patients do need it, because they collapse, they go home and for months they feel very bad and don’t know who to turn to.”*
***MID S2*** *: “The psychologist should be part of the team, both to help the women and to guide staff, because burnout does happen.”*
***NUR S3*** *: “We don’t know what techniques we can use to deal with conflicts that may arise during the clinical relationship.”*
Subcategory: influence of technology Verbatim
***OBST S13*** *: “We spend more time using technology than we do listening, looking into people’s eyes (...)”*
***NUR S3*** *: “An excess of technology can dehumanise the attention we provide.”*

*OBST* Obstetrician, *MID* Midwife, *NUR* Nurse, *NASST* Nursing assistant

## Discussion

As is apparent from our findings, although in health care it is frequently necessary to break bad news to patients, this obligation poses a major challenge to doctors and nurses, and can create difficult, painful situations [22, 23]. The way in which bad news is transmitted affects the patient’s understanding of the information received and hence the decisions taken in this respect, the psychological adaptation to new circumstances, participation in the process and any future changes made [24–26].

Difficulties may arise from communicators’ insecurity and anxiety, possibly due to inadequate training in communication techniques and care relations. Moreover, healthcare staff may lack the knowledge and skills needed to assess patients’ information needs and to motivate their active participation in decision making [15, 27–29].

In this respect, intuition and/or experience are not sufficient in themselves. Communication is not a gift but a skill that can be learned [30] and for which training must be provided, because it does not necessarily improve with experience [31]. The doctors, nurses and nursing assistants who took part in this study all agree that in developing their professional competence, they learned to communicate with patients by means of trial and error and by imitation, from observing the actions of colleagues with more experience. None of the medical workers taking part in our study had received a refresher course or specific training in this respect, a shortcoming that has also been reported in previous research [32–35].

Communication skills should be included as part of the training of healthcare personnel, together with the clinical competence specific to each branch of the profession [36]. Such training can enhance empathy in carers, helping them evaluate patients’ expectations, offer appropriate support, reduce emotional distress and foster compliance with clinical guidelines [26]. Indeed, good communication is an ethical and legal imperative [34, 37, 38].

In line with previous studies [33, 34, 39], we believe that other important aspects to be addressed include non-verbal language and the environment in which the bad news is to be communicated: this should be comfortable and quiet and enable privacy. However, this is not always the case, and organisational and structural problems have been identified. Consultation areas often fail to provide the above-mentioned characteristics, and noise, interruptions and lack of time due to the care burden all impede the creation of the therapeutic rapport between medical worker and patient that is necessary for successful collaboration between them.

Midwives, nurses and nursing assistants receive training in providing specific support to maternity patients, reinforcing personal qualities traditionally associated with their profession, such as closeness, kindness and sympathy. Corroborating the findings of earlier research, our results show that healthcare personnel are equipped with the necessary skills to produce a good nurse-patient relationship, although it has also been found that these capabilities are often lost if they are not refreshed during later professional practice [40].

While nurses must often play the role of communicators of bad news, many obstetricians consider this to be a function that is outside their sphere of competence. Nursing staff and midwives, however, are usually committed to working as a team, and the multidisciplinary approach has been shown to be the most effective means of communicating bad news. Indeed, if health teams do not function in a well-integrated way, the patient may receive differing or even contradictory information [33, 41].

To overcome barriers to communication, healthcare personnel should develop the ability to express empathy, closeness and solidarity with patients’ emotions, and also possess active listening skills – in areas such as paying careful attention and manifesting availability to help – together with self-assuredness, transmitting a sense of security on the basis of well-grounded opinions [3, 39, 42, 43].

The lack of training in the above skills to foster effective communication is often aggravated by an absence of feedback and by insufficient time to offer more support. As a result, the relationship with the patient is very limited despite the wish to provide high quality care. A possible problem in the carer-patient relation is the tendency of carers, in some cases, to (mis)interpret the wishes and needs of patients; in consequence, the response made may not meet the patient’s expectations, but correspond to what the carer believes appropriate. A good communicator should clarify matters with the patient, providing feedback to ensure that what is clear to one party is equally clear to the other [5, 6].

The acquisition of communication skills is hampered when there is no relationship between the learning process and the context in which the work must be carried out, when there is little or no flexibility in the scheduling of courses or workshops, when there is insufficient institutional recognition and when there are few opportunities for training to take place. Problems may also arise from a fear of becoming too involved when dealing with personal aspects such as feelings and emotions [44].

Health professionals may adopt different attitudes if they lack the skills to handle the emotional responses of patients who have received bad news. On the one hand, some carers believe that their responsibility is limited to addressing physical problems; in consequence, they will emphasise the development of skills related to the use of instruments developed in biomedicine, in order to provide a faster and more accurate diagnosis [1]. In monitoring the progress of a pregnancy control, such a carer’s entire attention could be focused on confirming or ruling out the presence of biological problems in the foetus. Accordingly, ultrasound examinations, screening tests, the signing of consent forms and entering medical records into computer files would occupy most of the time allocated to the consultation, leaving hardly any opportunity for a face-to-face exchange of views [10]. A carer may be highly skilled in the application of certain techniques, but this will be of little use if effective communication cannot be established [31]. In fact, technology should be auxiliary to the carer’s daily work [45]. Patient-centred care means understanding the human being from a biopsychosocial approach, recognising the need to consider not only the illness but also the patient’s personal experience, and to understand her as a person with emotions and private concerns (the psychological sphere) in multiple aspects of life, including work, family, and the partner (social sphere) [25, 26].

On the other hand, time pressures and limitations are sometimes cited to justify the lack of attention paid to psychosocial aspects of health care [10]. In contrast to this attitude among healthcare personnel, evidence suggests that patients strongly believe there is a need to investigate their unexpressed concerns, to teach them how to evaluate the information provided and to adopt appropriate measures, on the basis of personalised recommendations [46]. Our review of the literature and our analysis of the results obtained lead us to conclude that in a context in which patients can express their concerns and fears, motivated by the health carer’s open, sympathetic attitude, therapeutic communication can be established and the necessary emotional support supplied [47]. However, doctors and nurses do not usually consider the patient’s mood before communicating a diagnosis, or inquire about recent stressful episodes (such as the death of a loved one, or the presence of a severe illness in the patient or in a close relative), despite research evidence that the accumulation of stress-provoking experiences shortly before a traumatic event can increase the incidence of post-traumatic stress disorders [48].

Difficulties in communication may also be due to an unexpected diagnosis, without previous indications. When a diagnosis of this type must be confirmed, the health carer often experiences anxiety, a feeling of responsibility and a fear of censure, while being pressured to supply a rapid, convincing explanation and at the same time respond to the patient’s emotional reaction [8].

At other times, difficulty in transmitting an adverse diagnosis or a poor prognosis is the result of a heavy workload, together with pressing demands by patients and their families for information [49]. Studies have shown that when information is transmitted to patients and their families in sufficient quantity and quality, their anxiety is reduced and, in general, better and faster recovery is achieved and patient/carer collaboration is enhanced [23, 32].

Feelings of frustration and helplessness when the carer is unable to prevent, halt or reverse a negative outcome also hamper communication, especially when the therapeutic options are limited or non-existent. The carer must then seek to provide comfort in a situation that does not offer grounds for being hopeful [26, 50]. Feelings of frustration and powerlessness may be compounded by legal concerns, with the worry that an unsatisfied patient may present an official complaint. The judicialisation of healthcare issues may generate the notion that every human being has the right to be healed and that any failure in this respect must be due to an error, which should be punished. In the health service, more complaints are made regarding the quality of information received than any other aspect of health care [10]. Moreover, the provision of informed consent does not always guarantee the reality of bidirectional communication, but may serve only as a legal safeguard [51].

Our results also show that some healthcare professionals, due to the experience of patients’ suffering and to taboos regarding death, erect barriers to communication through automated responses and patterns of avoidance, especially when there is the possibility of transferring responsibility to other carers [52]. This type of behaviour may arise due to a lack of support system for the healthcare team, or to the absence or outdated status of action protocols [50].

Language and cultural differences are the most common types of communication barrier, due to the multilingual and multicultural nature of the treatment population [53]. The two most important causes of ineffective communication are the precarity of information provided to patients and the absence of comprehension [54, 55].

The link between the principal investigator and the institution in which the study was performed may be regarded as a limitation of this study, insofar as it may have influenced the interpretation of certain professional practices. On the other hand, this association facilitated access to a wide range of scenarios in which patients and carers interact, thus providing an authentic outlook on healthcare practice.

Another potential issue is that, given the inherent properties of the qualitative method applied and the local nature of this study, the findings obtained might not be readily extrapolated to other contexts (although the method used can be transferred without difficulty). The effect of subjectivity means that a given phenomenon may be perceived, interpreted and experienced differently from one individual to another. Therefore, the interpretations of our participants, regarding the specific problem addressed in this study, will not necessarily be shared by other professionals, interacting with patients in different contexts.

## Conclusions

The increasing dependence of certain diagnostic procedures on technological resources may be detrimental to interpersonal relationships, making them cold and distant. For healthcare personnel, the human quality of their profession has deteriorated, mainly due to the heavy caseloads experienced, which increasingly limit the time that can be spent with each patient. In order to improve communication, more attention should be paid to the human and spiritual dimensions of healthcare, giving greater weight to empathy, authenticity and listening (without imposing one’s own interpretation). The analysis performed leads us to draw the following conclusions: a different model of clinical relationship should be promoted, based on shared decision making, and greater clarity should be granted to the functions of the multidisciplinary team with respect to the patient’s grieving when a pregnancy is interrupted. To achieve these goals, protocols should be implemented to ensure comprehensive care provision, addressing not only the biological sphere but also psychosocial concerns. In this respect, too, specific training should be provided, at undergraduate and postgraduate levels, in social skills and cultural competence. In short, this study identifies possible areas of improvement related to the interventions of healthcare personnel and to the organisation of the institution itself, with particular respect to the communication to patients of an adverse prenatal diagnosis.

## Footnotes

1 The superscripts correspond to the authors who have made this part of the methodology.

## References

[1] M. Rodriguez de Alba, A. Bustamante-Aragones, S. Perlado, M. J. Trujillo-Tiebas, J. Diaz-Recasens, J. Plaza-Arranz, y C. Ramos, Noninvasive prenatal diagnosis of monogenic disorders. Expert Opin Biol Ther, vol. 12 Suppl 1, pp. S171-S179, jun. 2012. 10.1517/14712598.2012.674509 22507053

[2] L. Butalid, J. M. Bensing, y P. F. M. Verhaak, «Talking about psychosocial problems: an observational study on changes in doctor-patient communication in general practice between 1977 and 2008.», Patient Educ Couns, vol. 94, n.^o^ 3, pp. 314-321, mar. 2014. 10.1016/j.pec.2013.11.004 24360508

[3] F. A. Derksen, T. C. Olde Hartman, J. M. Bensing, y A. L. Lagro-Janssen, «Managing barriers to empathy in the clinical encounter: a qualitative interview study with GPs.», The British journal of general practice : the journal of the Royal College of General Practitioners, vol. 66, n.^o^ 653, pp. e887-e895, dic. 2016. 10.3399/bjgp16X687565 PMC5198671 27884917

[4] R. M. Epstein y R. L. J. Street, «Shared mind: communication, decision making, and autonomy in serious illness.», Ann Fam Med, vol. 9, n.^o^ 5, pp. 454-461, 2011. 10.1370/afm.1301 PMC3185482 21911765

[5] R. L. J. Street, G. Makoul, N. K. Arora, y R. M. Epstein, «How does communication heal? Pathways linking clinician-patient communication to health outcomes.», Patient Educ Couns, vol. 74, n.^o^ 3, pp. 295-301, mar. 2009. 10.1016/j.pec.2008.11.015 19150199

[6] J. K. Rao, L. A. Anderson, T. S. Inui, y R. M. Frankel, «Communication interventions make a difference in conversations between physicians and patients: a systematic review of the evidence.», Med Care, vol. 45, n.^o^ 4, pp. 340-349, abr. 2007. 10.1097/01.mlr.0000254516.04961.d5 17496718

[7] O. Barr, H. Skirton, «Informed decision making regarding antenatal screening for fetal abnormality in the United Kingdom: a qualitative study of parents and professionals.», Nursing & health sciences, vol. 15, n.^o^ 3, pp. 318-325, sep. 2013. 10.1111/nhs.12034 23347127

[8] A. L. Greiner, J. Conklin, «Breaking bad news to a pregnant woman with a fetal abnormality on ultrasound.», Obstetrical & gynecological survey, vol. 70, n.^o^ 1, pp. 39-44, ene. 2015. 10.1097/OGX.0000000000000149 25616346

[9] J. Hodgson, P. Pitt, S. Metcalfe, J. Halliday, M. Menezes, J. Fisher, C. Hickerton, K. Petersen, y B. McClaren, «Experiences of prenatal diagnosis and decision-making about termination of pregnancy: a qualitative study.», Aust N Z J Obstet Gynaecol, vol. 56, n.^o^ 6, pp. 605-613, dic. 2016. 10.1111/ajo.12501 27402530

[10] R. Simpson y R. Bor, «‘I’m not picking up a heart-beat’: experiences of sonographers giving bad news to women during ultrasound scans.», The British journal of medical psychology, vol. 74 Part 2, pp. 255-272, jun. 2001. 11453176

[11] R.-L. Van Keer, R. Deschepper, A. L. Francke, L. Huyghens, y J. Bilsen, «Conflicts between healthcare professionals and families of a multi-ethnic patient population during critical care: an ethnographic study.», Critical care (London, England), vol. 19, p. 441, dic. 2015. 10.1186/s13054-015-1158-4 PMC4699338 26694072

[12] K. Sweeny, J. A. Shepperd, y P. K. J. Han, «The goals of communicating bad news in health care: do physicians and patients agree?», Health expectations : an international journal of public participation in health care and health policy, vol. 16, n.^o^ 3, pp. 230-238, sep. 2013. 10.1111/j.1369-7625.2011.00709.x PMC5060662 21771225

[13] L. Fallowfield y V. Jenkins, «Communicating sad, bad, and difficult news in medicine.», Lancet (London, England), vol. 363, n.^o^ 9405, pp. 312-319, ene. 2004. 10.1016/S0140-6736(03)15392-5 14751707

[14] R. R. Wallace, S. Goodman, L. R. Freedman, V. K. Dalton, y L. H. Harris, «Counseling women with early pregnancy failure: utilizing evidence, preserving preference.», Patient Educ Couns, vol. 81, n.^o^ 3, pp. 454-461, dic. 2010. 10.1016/j.pec.2010.10.031 21093193

[15] J. Halpern, «Empathy and patient-physician conflicts.», J Gen Intern Med, vol. 22, n.^o^ 5, pp. 696-700, may 2007. 10.1007/s11606-006-0102-3 PMC1852904 17443382

[16] L. Martin, J. T. Gitsels-van der Wal, M. T. R. Pereboom, E. R. Spelten, E. K. Hutton, y S. van Dulmen, «Clients’ psychosocial communication and midwives’ verbal and nonverbal communication during prenatal counseling for anomaly screening.», Patient Educ Couns, vol. 99, n.^o^ 1, pp. 85-91, ene. 2016. 10.1016/j.pec.2015.07.020 26298217

[17] M. Driessnack, V. D. Sousa, y I. A. C. Mendes, «An overview of research designs relevant to nursing: part 2: qualitative research designs.», Rev Lat Am Enfermagem, vol. 15, n.^o^ 4, pp. 684-688, 2007. 10.1590/s0104-11692007000400025 17957836

[18] Guba EG, Lincoln YS. Competing paradigms in qualitative research. Thousand Oaks: The handbook of qualitative research. CA: Sage; 1994.

[19] M. Pla, "Rigor in qualitative research", Aten Primaria, vol. 24, n.^o^ 5, pp. 295-300, 1999. 10590563

[20] Taylor R, Bogdan S.J (1987). Introduction to qualitative research methods. The search for meanings.

[21] N. Carter, D. Bryant-Lukosius, A. DiCenso, J. Blythe, y A. J. Neville, «The use of triangulation in qualitative research.», Oncol Nurs Forum, vol. 41, n.^o^ 5, pp. 545-547, sep. 2014. 10.1188/14.ONF.545-547 25158659

[22] D. Meitar, O. Karnieli-Miller, y S. Eidelman, «The impact of senior medical students’ personal difficulties on their communication patterns in breaking bad news.», Academic medicine : journal of the Association of American Medical Colleges, vol. 84, n.^o^ 11, pp. 1582-1594, nov. 2009. 10.1097/ACM.0b013e3181bb2b94 19858822

[23] F. Delevallez, A. Lienard, A.-S. Gibon, y D. Razavi, «[Breaking bad news in oncology: the Belgian experience].», Revue des maladies respiratoires, vol. 31, n.^o^ 8, pp. 721-728, oct. 2014. 10.1016/j.rmr.2014.07.003 25391507

[24] C. Burgers, C. J. Beukeboom, y L. Sparks, «How the doc should (not) talk: when breaking bad news with negations influences patients’ immediate responses and medical adherence intentions.», Patient Educ Couns, vol. 89, n.^o^ 2, pp. 267-273, nov. 2012. 10.1016/j.pec.2012.08.008 22938871

[25] A. Gesser-Edelsburg y N. A. E. Shahbari, «Decision-making on terminating pregnancy for Muslim Arab women pregnant with fetuses with congenital anomalies: maternal affect and doctor-patient communication.», Reprod Health, vol. 14, n.^o^ 1, p. 49, abr. 2017. 10.1186/s12978-017-0312-7 PMC5379523 28376917

[26] A. Herrera, M. Rios, J. M. Manriquez, y G. Rojas, «[Breaking bad news in clinical practice].», Rev Med Chil, vol. 142, n.^o^ 10, pp. 1306-1315, oct. 2014. 10.4067/S0034-98872014001000011 25601116

[27] N. H. M. Labrie y P. J. Schulz, «Exploring the relationships between participatory decision-making, visit duration, and general practitioners’ provision of argumentation to support their medical advice: results from a content analysis.», Patient Educ Couns, vol. 98, n.^o^ 5, pp. 572-577, may 2015. 10.1016/j.pec.2015.01.017 25746127

[28] J. G. Lalor, D. Devane, y C. M. Begley, «Unexpected diagnosis of fetal abnormality: women’s encounters with caregivers.», Birth (Berkeley, Calif), vol. 34, n.^o^ 1, pp. 80-88, mar. 2007. 10.1111/j.1523-536X.2006.00148.x 17324182

[29] M. Brann y J. J. Bute, «Communicating to promote informed decisions in the context of early pregnancy loss.», Patient Educ Couns, vol. 100, n.^o^ 12, pp. 2269-2274, dic. 2017. 10.1016/j.pec.2017.06.016 28645640

[30] J. Shaw, R. Brown y S. Dunn, «The impact of delivery style on doctors’ experience of stress during simulated bad news consultations.», Patient Educ Couns, vol. 98, n^°^ 10, pp. 1255-1259, oct. 2015. 10.1016/j.pec.2015.08.023 26320824

[31] P. M. Moore, S. Rivera Mercado, M. Grez Artigues, y T. A. Lawrie, «Communication skills training for healthcare professionals working with people who have cancer.», The Cochrane database of systematic reviews, no. 3, p. CD003751, mar. 2013. 10.1002/14651858.CD003751.pub3 PMC6457800 23543521

[32] A. P. Jacques, E. J. Adkins, S. Knepel, C. Boulger, J. Miller, y D. P. Bahner, «Educating the delivery of bad news in medicine: Preceptorship versus simulation.», International journal of critical illness and injury science, vol. 1, n.^o^ 2, pp. 121-124, jul. 2011. 10.4103/2229-5151.84796 PMC3249843 22229135

[33] M. L. Bascunan, «[Truth disclosure in medicine: psychological perspective].», Rev Med Chil, vol. 133, n.^o^ 6, pp. 693-698, jun. 2005. 10.4067/s0034-98872005000600012 16075136

[34] W. F. Baile, R. Buckman, R. Lenzi, G. Glober, E. A. Beale, y A. P. Kudelka, «SPIKES-A six-step protocol for delivering bad news: application to the patient with cancer.», Oncologist, vol. 5, n.^o^ 4, pp. 302-311, 2000. 10.1634/theoncologist.5-4-302 10964998

[35] K. R. Monden, L. Gentry, y T. R. Cox, «Delivering bad news to patients.», Proc (Baylor Univ Med Cent), vol. 29, n.^o^ 1, pp. 101-102, ene. 2016. 10.1080/08998280.2016.11929380 PMC4677873 26722188

[36] D. Armentrout y L. A. Cates, «Informing parents about the actual or impending death of their infant in a newborn intensive care unit.», The Journal of perinatal & neonatal nursing, vol. 25, n.^o^ 3, pp. 261-267, 2011. 10.1097/JPN.0b013e3182259943 21825916

[37] C. Strong, «Fetal anomalies: ethical and legal considerations in screening, detection, and management.», Clin Perinatol, vol. 30, n.^o^ 1, pp. 113-126, mar. 2003. 10.1016/s0095-5108(02)00083-0 12696790

[38] A. Arber y A. Gallagher, «Breaking bad news revisited: the push for negotiated disclosure and changing practice implications.», Int J Palliat Nurs, vol. 9, n.^o^ 4, pp. 166-172, abr. 2003. 10.12968/ijpn.2003.9.4.11497 12734453

[39] S. Nunez, T. Marco, G. Burillo-Putze, y J. Ojeda, «[Procedures and skills for the communication of bad news in emergency units].», Med Clin, vol. 127, n.^o^ 15, pp. 580-583, oct. 2006. 10.1157/13094002 17145016

[40] R. Norouzinia, M. Aghabarari, M. Shiri, M. Karimi, y E. Samami, «Communication barriers perceived by nurses and patients.», Global journal of health science, vol. 8, n.^o^ 6, pp. 65-74, sep. 2015. 10.5539/gjhs.v8n6p65 PMC4954910 26755475

[41] H. Gluyas, «Effective communication and teamwork promotes patient safety.», Nursing standard (Royal College of Nursing (Great Britain) : 1987), vol. 29, n.^o^ 49, pp. 50-57, ago. 2015. 10.7748/ns.29.49.50.e10042 26243123

[42] M. Omura, J. Maguire, T. Levett-Jones, T. E. Stone, «Effectiveness of assertive communication training programs for health professionals and students: a systematic review protocol.», JBI database of systematic reviews and implementation reports, vol. 14, n.^o^ 10, pp. 64-71, oct. 2016. 10.11124/JBISRIR-2016-003158 27846116

[43] A. Lyndon, M. G. Zlatnik, y R. M. Wachter, «Effective physician-nurse communication: a patient safety essential for labor and delivery.», Am J Obstet Gynecol, vol. 205, n.^o^ 2, pp. 91-96, ago. 2011. 10.1016/j.ajog.2011.04.021 PMC3219810 21640970

[44] M. Schmid Mast, A. Kindlimann, y W. Langewitz, «Recipients’ perspective on breaking bad news: how you put it really makes a difference.», Patient Educ Couns, vol. 58, n.^o^ 3, pp. 244-251, sep. 2005. 10.1016/j.pec.2005.05.005 16081235

[45] F. Oflaz y H. Vural, «The evaluation of nurses and nursing activities through the perceptions of inpatients.», Int Nurs Rev, vol. 57, n.^o^ 2, pp. 232-239, jun. 2010. 10.1111/j.1466-7657.2009.00772.x 20579159

[46] G. Novick, «Women’s experience of prenatal care: an integrative review.», Journal of midwifery & women’s health, vol. 54, n.^o^ 3, pp. 226-237, 2009. 10.1016/j.jmwh.2009.02.003 PMC2754192 19410215

[47] E. C. Heberlein, A. H. Picklesimer, D. L. Billings, S. Covington-Kolb, N. Farber, y E. A. Frongillo, «Qualitative comparison of Women’s perspectives on the functions and benefits of group and individual prenatal care.», Journal of midwifery & women’s health, vol. 61, n.^o^ 2, pp. 224-234, 2016. 10.1111/jmwh.12379 26878599

[48] J. C. M. Cole, J. S. Moldenhauer, K. Berger, M. S. Cary, H. Smith, V. Martino, N. Rendon, y L. J. Howell, «Identifying expectant parents at risk for psychological distress in response to a confirmed fetal abnormality.», Archives of women’s mental health, vol. 19, n.^o^ 3, pp. 443-453, jun. 2016. 10.1007/s00737-015-0580-6 26392365

[49] L. T. de Beer, J. Pienaar, y S. J. Rothmann, «Work overload, burnout, and psychological ill-health symptoms: a three-wave mediation model of the employee health impairment process.», Anxiety Stress Coping, vol. 29, n.^o^ 4, pp. 387-399, jul. 2016. 10.1080/10615806.2015.1061123 26079200

[50] S. Newell y Z. Jordan, «The patient experience of patient-centered communication with nurses in the hospital setting: a qualitative systematic review protocol.», JBI database of systematic reviews and implementation reports, vol. 13, n.^o^ 1, pp. 76-87, ene. 2015. 10.11124/jbisrir-2015-1072 26447009

[51] G. Ferreira Padilla, T. Ferrandez Anton, y J. Baleriola Julvez, «[Informed consent versus “communicated consent” in the need to ensure a two-way doctor-patient relationship and to improve patient and doctor satisfaction].», Aten Primaria, vol. 45, n.^o^ 4. Spain, pp. 225-226, abr-2013. 10.1016/j.aprim.2012.11.007 PMC6983581 23337465

[52] F. Baena-Antequera, y E. Jurado-Garcia, «[The woman at the termination of pregnancy for fetal anomalies: clinical case].», Enferm Clin, vol. 25, n.^o^ 5, pp. 276-281, 2015. 10.1016/j.enfcli.2015.07.003 26342815

[53] E. Paternotte, F. Scheele, C. M. Seeleman, L. Bank, A. J. J. A. Scherpbier, y S. van Dulmen, «Intercultural doctor-patient communication in daily outpatient care: relevant communication skills.», Perspectives on medical education, vol. 5, n.^o^ 5, pp. 268-275, oct. 2016. 10.1007/s40037-016-0288-y PMC5035277 27638395

[54] H. Harmsen, L. Meeuwesen, J. van Wieringen, R. Bernsen, y M. Bruijnzeels, «When cultures meet in general practice: intercultural differences between GPs and parents of child patients.», Patient Educ Couns, vol. 51, n.^o^ 2, pp. 99-106, oct. 2003. 10.1016/s0738-3991(02)00195-7 14572938

[55] R. L. Sudore, C. S. Landefeld, E. J. Perez-Stable, K. Bibbins-Domingo, B. A. Williams, y D. Schillinger, «Unraveling the relationship between literacy, language proficiency, and patient-physician communication.», Patient Educ Couns, vol. 75, n.^o^ 3, pp. 398-402, jun. 2009. 10.1016/j.pec.2009.02.019 PMC4068007 19442478

